# Global research trends in palliative care for breast cancer from 2012 to 2022: A scientometric analysis

**DOI:** 10.3389/fonc.2023.1104531

**Published:** 2023-02-23

**Authors:** Yixian Sun, Jinyao Wu, Huiting Tian, Xiuhua Qiu, Ying Fang, Yingjie Xiao, Jiehua Zheng, Yanqiong Zhou

**Affiliations:** ^1^ Department of Thyroid, Breast, and Hernia Surgery, General Surgery, The Second Affiliated Hospital of Shantou University Medical College, Shantou, Guangdong, China; ^2^ Department of Educational Administration Office/Humanistic Education, Shantou University Medical College, Guangdong, China; ^3^ Department of Mental Health and Counseling Center, Shantou University Medical College, Guangdong, China; ^4^ Department of Medical Humanities, Shantou University Medical College, Guangdong, China; ^5^ Department of Pharmacy, The Second Affiliated Hospital of Shantou University Medical College, Shantou, Guangdong, China

**Keywords:** palliative care, breast cancer, scientometrics, bibliometrix, VOSviewer

## Abstract

**Purpose:**

We used bibliometric methods to evaluate the global scientific output of palliative care breast cancer research and to explore the current status and further research directions in the field over the past decade.

**Methods:**

All relevant publications from the year 2012 to 2022 were retrieved from Web of Science. We applied VOSviewer and Bibliometrix R v4.2.1 to obtain information on subject domains, annual publication output and citations, countries and authors with the highest productivity, influential journals and articles, and popular keywords.

**Results:**

In total, 1529 publications were included in the final analysis. Health services and supportive care, pain and symptom management were the focus of the research in the field. From the year 2017 to 2021, the annual publication output was abundant and peaked in 2018. The systematic review by Fitzmaurice et al. in 2017 was the most-cited publication. The United States was the leading country with the maximum number of publications, citations, and link strengths with other countries. The most contributing institution was University of Toronto. E. Bruera was the most prolific author, ranking first among the authors in both the H and M index. The journal with the most publications was *Palliative & Supportive Care*. Keywords analysis indicated that exploring the significant degree of palliative care to reduce anxiety and depression in breast cancer patients may be a good research direction. In addition, how to assess the optimal timing of palliative care interventions and further exploring the specific contradiction between insufficient medical resources and palliative care are also possible research directions.

**Conclusion:**

Palliative care plays an important role in the treatment of breast cancer. With the help of a scientometric analysis in this field, researchers can clarify the current research status and hotspots worth fully exploring.

## 1 Introduction

In the year 2020, breast cancer supplanted lung cancer as the most diagnosed cancer worldwide (1). The increasing incidence of breast cancer is closely related to human development, with the same trend expected in regions of the world that are currently undergoing economic transition (2). The incidence of female breast cancer is higher in countries with a higher socioeconomic status, whereas mortality rates and disability-adjusted life years are higher in poorly developed countries (3). The disease burden of breast cancer includes physical, psychological, social, and financial dimensions (4) that are related not only to the disease itself but also to its treatment, including aesthetic results, effects on sexual activity, and psychosocial impact. These aspects affect patients’ quality of life during and after treatment or at relapse (5). Patients’ suffering can be alleviated by integrating palliative care into standard breast cancer treatment programs.

Palliative care involves using a multidisciplinary approach to relieve physical pain and psychological symptoms in order to provide a support system for patients that improves their quality of life until the end of life and help the family cope with their grief (6). Palliative care services have been integrated into traditional medical models; in 2014, the World Health Assembly proposed the incorporation of palliative care into existing healthcare systems (7). According to a comprehensive care model, palliative care is most effective when incorporated early on (ie, from the time of diagnosis) in the cancer management program along with anticancer therapy (8). Early implementation of specialized palliative care services for patients with advanced cancer has been shown to improve patients’ quality of life, increase their satisfaction with care, and mitigate depression (9–11). Given these benefits, palliative care is increasingly being incorporated into standard oncology care programs (12).

Although there is a substantial amount of research on palliative care for breast cancer, there have been no reports on the current state of knowledge in this field. We therefore conducted a bibliometric analysis of the most productive countries and authors, influential journals, hot topics, and keywords of relevant articles in order to determine the current state of and future directions in breast cancer palliative care research.

## 2 Materials and methods

### 2.1 Data collection

We searched all publications related to palliative care and breast cancer from 2012 to 2022 in the Web of Science core database, including Science Citation Index-EXPANDED, Social Sciences Citation Index, and Arts & Humanities Citation Index (all 2003 to present); Emerging Sources Citation Index (2017 to present); Current Chemical Reactions-EXPANDED (1985 to present); and Index Chemicus (1993 to present). The Medical Subject Headings and entry terms “palliative care” and “breast cancer” were used as search strategies. Retrieval queries included the following: #1, ALL=(“Palliative Care”) OR ALL=(“Palliative Treatment*”) OR ALL=(“Palliative Therapy”) OR ALL=(“Palliative Supportive Care”) OR ALL=(“Palliative Surgery”); #2, ALL=(“breast cancer”) OR ALL=(“Breast Neoplasm”) OR ALL=(“Breast Tumor*”) OR ALL=(“Mammary Cancer*”) OR ALL=(“Malignant Neoplasm of Breast”) OR ALL=(“Breast Malignant Neoplasm*”) OR ALL=(“Breast Malignant Tumor*”) OR ALL=(“Mammary Carcinoma, Human”) OR ALL=(“Mammary Neoplasms, Human”) OR ALL=(“Breast Carcinoma*”); “#3”, “#1”, and “#2”. Also, the timespan of these publications was then filtered from 2012 to 2022. The search was conducted on July 10, 2022 and yielded 1654 articles. We set the document type to article or review article, restricted the language to English, and excluded one retracted publication. This yielded 1529 publications including 1134 articles (74.17%) and 395 review articles (25.83%); meanwhile, 125 publications were excluded including 69 non-English documents, 38 early access articles, 16 proceedings papers, 1 book chapter, and 1 retracted publication.

### 2.2 Data analysis

We initially used the online “analyze results” function of Web of Science to obtain information on the year of publication, document type, area of research, authors, affiliations, journal, publisher, countries, language, funding agencies, and open access. Other information such as the number of all citations (excluding self-citations), sum of references (excluding self-citations), average citations per item, and the H index were also obtained with the Web of Science’s Citation Report function. We imported the information collected in these publications including title, authors, affiliations, language, article type, abstract, keywords, and cited references into Biblioshiny (Bibliometrix’s network interface) and VOSviewer.

Bibliometrix (R v4.2.1) is an open-source research tool for scientific and bibliometric quantitative analyses of Web of Science data and visual representation of the results (13). By importing the original files of retrieved data into the Biblioshiny website, we obtained information for these publications including time span, number of sources, number of articles, number of references, file type, file content, authors and co-authors. With this information, we made a preliminary judgment on whether the results met the inclusion criteria. Other information that was collected included annual scientific output, average annual article citations, contributing countries, contributing institutions, and keywords.

VOSviewer v1.6.17 is a network analysis software for analyzing the most cited literature and countries of co-authors, and for reference and keyword co-occurrence network analysis (14). Ethics approval was not required for this study.

## 3 Results

### 3.1 Subject domain analysis

Up to 2022, there were 1529 publications in the field of palliative care for breast cancer. The topics covered by these publications included “health service and supportive care”, “psychosocial”, “symptom and symptom management”, “pain and pain management”, “nursing”, “quality of life”, and “health policy and environment”. **Table 1** lists the subject domains in this field and the number of times each subject was discussed in the publications. There were 305 articles pertaining to health services and supportive care, 279 related to psychosocial aspects of breast cancer patients who received palliative care, 255 on symptoms and symptom management, 206 on pain and pain management, 224 on nursing, 177 on quality of life, and 73 on health policy and environment. The subject domains provided an overview of the research focus; a total of 766 publications were related to health services and supportive care as well as pain and symptom management. Publications pertaining to psychosocial aspects of breast cancer patients who received palliative care, which was also the focus of many studies, accounted for 18.25% of all subject domains.

**Table 1:** Subject domains in the publications in the field of palliative care in breast cancer.

Subject Domains	Publications
Health services&supportive care	315
Psychosocial	279
Symptoms and symptom management	255
Pain and pain management	206
Nursing	224
Quality of life	177
Health policy&environment	73

### 3.2 Annual publication output trends and citation analysis

**Figure 1:**
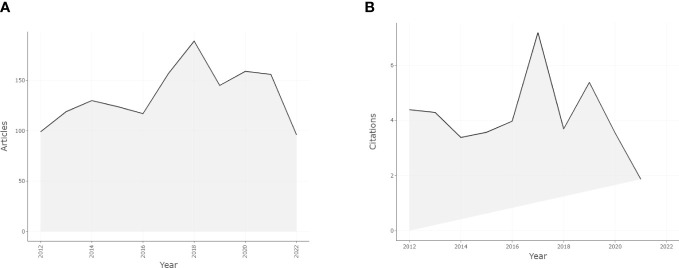
Annual scientific production and average article citations per year from 2012 to 2022 in this research field. **(A)** Annual scientific production from 2012 to 2022 in this research field. **(B)** Average article citations per year from 2012 to 2022 in this research field.

We retrieved 1529 articles related to palliative care for breast cancer that were published in the past 10 years from Web of Science. The year with the most publications was 2018 (n=189, 12.36%). Starting from 2012 when 99 articles (6.47%) were published, there was a year-over-year increase in the number of publications, although the output was relatively stable between 2019 and 2021 (**Figure 1A**). There was a rapid increase in the number of publications from 2016 to 2018, with the rate reaching a peak in 2017 (34.19%). A. Ahmad discussed the trends in incidence and mortality of breast cancer in the United States (US) from 2009 through 2018 while also providing an overview of recently reported global estimates (15). The incidence rate increased during this period, especially from 2016 to 2018; in 2016, the American Society of Clinical Oncology guideline was updated with a provisional clinical opinion on the integration of palliative care into standard oncology care (16). These 2 factors may account for the surge in publications since 2016. The median annual growth rate was 9.24% and was highest in 2017 (34.19%) and lowest in 2019 (−23.28%).

**Figure 2:**
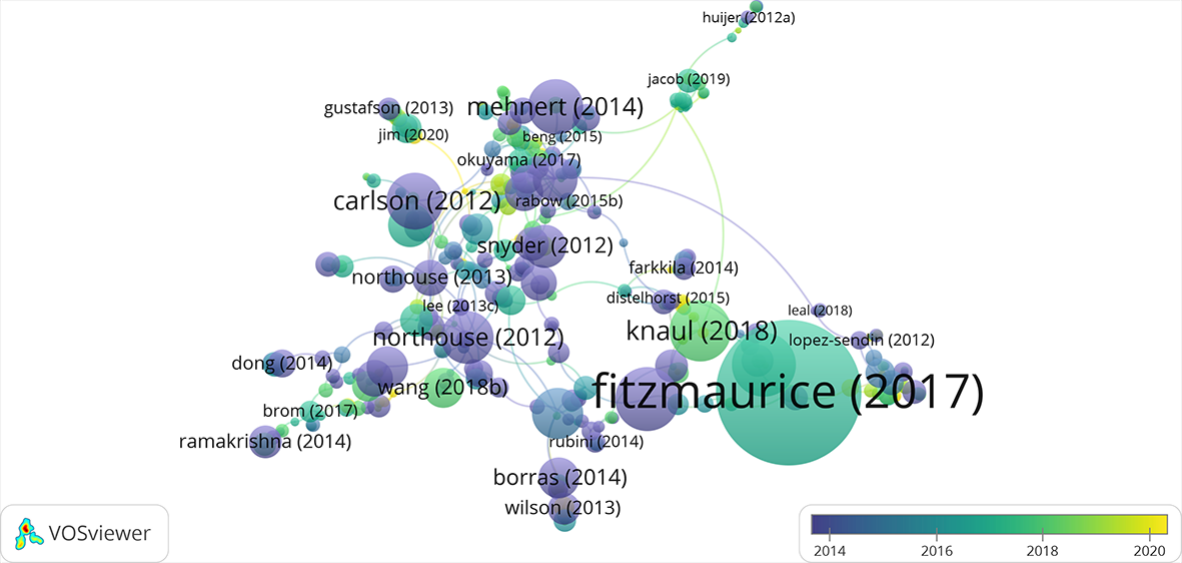
Network map of citation between documents with more than 5 citations.

Citation analysis is a bibliometric method that uses citation rates to evaluate research performance. The number of citations of an article reflects its scientific importance (17). The total number of references in the retrieved articles was 26 159, including 538 self-citations and 25 621 non–self-citations (97.94% of all references). Collectively, the retrieved articles were cited 30 622 times, of which 29 633 (96.77%) were non–self-citations. The number of citations rose from 84 in 2012 to 3463 in 2020. The average time to citation for each article was 20.03. The average number of citations per year from 2012 to 2021 is shown in **Figure 1B**; publications from 2017 were most frequently cited (7.2), with the trend declining since 2012 and fluctuating since 2016. The articles cited in the field of breast cancer palliative care research are represented graphically in **Figure 2**; a larger circle represents more citations and the distance between circles represents the strength of their association according to their co-citation frequency.

Highly cited articles revealed research hotspots. All of the studies were published between 2012 and 2019. The systematic review by Fitzmaurice et al. was cited 2399 times, and concluded that breast cancer management strategies should focus on early detection in addition to effective treatment and target the full spectrum of care including surgical, medical, and radiation oncology; affordable chemotherapeutics and supportive drugs; and survivorship support and palliative and hospice care (18). The study by Palma et al. (19) and review by Ginsburg et al. (20) were cited 812 and 457 times, respectively. These articles described the burden of breast cancer and highlighted the urgent need for sustainable investments in the entire continuum of cancer management from prevention to palliative care and in the development of high-quality population-based cancer registries in low- and middle-income countries. There were 35 studies that were cited over 100 times, collectively accounting for 2.29% of all citations.

### 3.3 Analysis of countries and institutions

**Figure 3:**
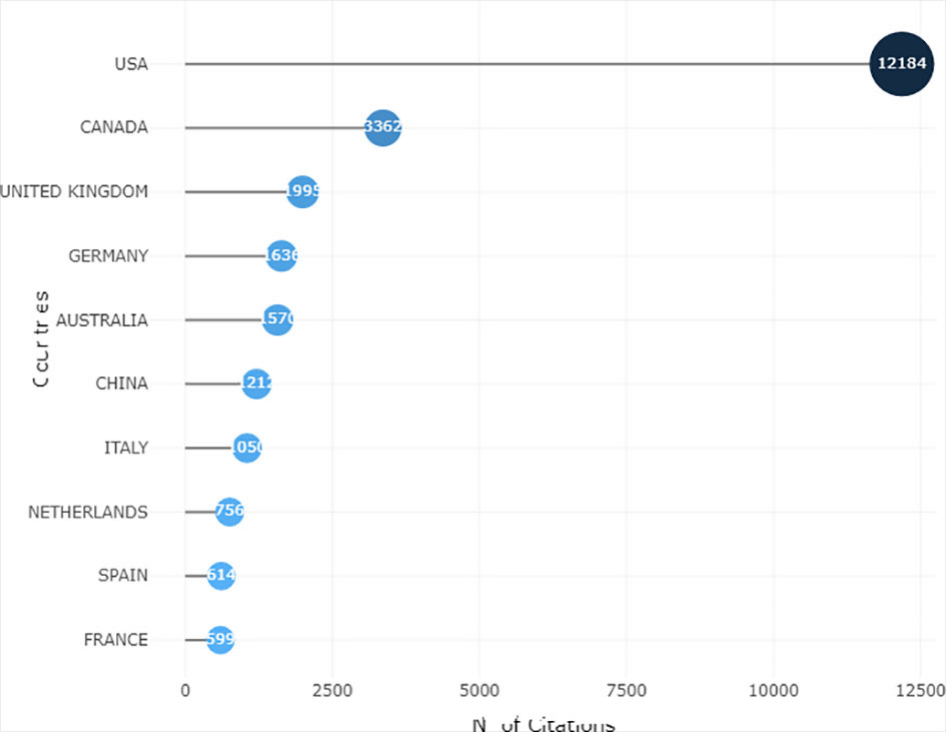
Top 10 cited countries contributing to this research area.

Of the 1529 documents, 70 countries and regions with a range of gross domestic products (GDPs) contributed to research on palliative care for breast cancer. According to the latest data released by the World Bank, the top 10 countries in the GDP rankings (current US$) in 2021 were the US, China, Japan, Germany, United Kingdom (UK), India, France, Italy, Canada, and Republic of Korea. Countries with high GDP (eg, US, China, and UK) also had high publication output and more citations. **Table 2** lists the number of articles published in the 10 countries with the highest research productivity. The US had the highest output with 389 published studies (25.44% of the total), followed by the UK (n=94, 6.15%), China (n=93, 6.08%), Germany (n=82, 5.36%), and Canada (n=81, 5.30%). **Figure 3** lists the 10 countries with the highest number of citations. Publications from the US were the most frequently cited (n=12 184), followed by those from Canada (n=3362), UK (n=1995), Germany (n=1636), and Australia (n=1570).

**Table 2:** Top 10 productive countries contributing to this research area.

Country	Number of Articles Published
USA	389
China	93
The United Kingdom	94
Canada	81
Germany	82
India	59
Australia	79
Jpan	55
Italy	65
Netherlands	44

**Figure 4:**
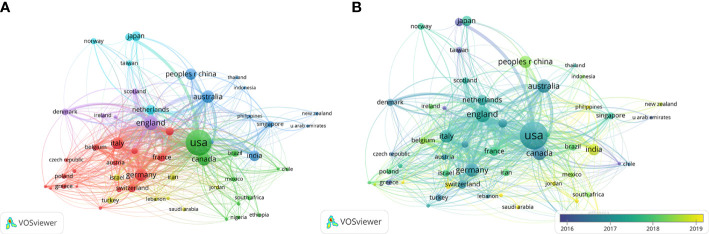
Co-authorship analysis of countries in this field. **(A)** Network map of co-authorship between countries with more than five publications. **(B)** Overlay map of co-authorship between countries with more than five publications. (The blue color represents the earlier years; the yellow color represents the more recent years.).

The co-authorship analysis included 55 countries with more than 5 publications in the research area; the results are represented graphically in **Figure 4A**. When ordered according to the total link strength, the top 5 countries were the US (total link strength=327), England (total link strength=265), Italy (total link strength=212), Germany (total link strength=153), and France (total link strength=150). The size of the circle in the figure reflects the total link strength of different countries, and the distance between circles represents the strength of the link according to co-authorship frequency. Over time, some developing countries increased their research productivity in the area of palliative care for breast cancer including Brazil (total link strength=63), India (total link strength=63), Poland (total link strength=55), China (total link strength=46), and Mexico (total link strength=42) (**Figure 4B**). Countries with the highest GDP such as US, England, Italy, Germany, Canada, and Australia also had high research productivity and total link strength.

A total of 2686 institutions contributed to the field (**Table 3**). University of Toronto had the most publications (n=84, 2.22%), followed by University of Texas MD Anderson Cancer Center (n=70, 1.94%), Dana-Farber Cancer Institute (n=50, 1.94%), Memorial Sloan-Kettering Cancer Center (n=47, 1.89%), and University of Washington (n=45, 1.8,4%). We analyzed co-authorship at institutions with more than 5 publications and obtained results for 196; the top 5 were University of Toronto (total link strength=93), Dana-Farber Cancer Institute (total link strength=90), Harvard Medical School (total link strength=88), University of Washington (total link strength=88), and Massachusetts General Hospital (total link strength=86) (**Table 4**).

**Table 3:** Most productive institutions contributing to this research field.

Institution	Articles
University of Toronto	84
University of Texas MD Anderson Cancer Center	70
Dana-Farber Cancer Institute	50
Memorial Sloan-Kettering Cancer Center	47
University of Washington	45
Harvard Medical School	44
University of Sydney	44
University of Pittsburgh	39
Massachusetts General Hospital	37
University of Western Australia	35

**Table 4:** Institutions with the most total link strength contributing to this research field.

Institution	Total link strength
University of Toronto	93
Dana-Farber Cancer Institute	90
Harvard Medical School	88
University of Washington	88
Massachusetts General Hospital	86

### 3.4 Journal analysis

The 1529 articles were published in 495 journals; the 10 most highly represented journals, which are focused on palliative care, are shown in **Table 5**. Collectively, the top 10 journals published 485 articles, accounting for 31.72% of the total. *Palliative & Supportive Care* published the most articles (n=109), followed by *Supportive Care in Cancer* (n=73), *Current Opinion in Supportive and Palliative Care* (n=62), *Indian Journal of Palliative Care* (n=48), *BMJ Supportive & Palliative Care* (n=42), *Journal of Pain and Symptom Management* (n=35), and *Psycho-Oncology* (n=35). **Table 6** lists citations of the top 10 journals publishing in this research area. *Journal of Clinical Oncology* had the most citations (n=3977 references), followed by *Psycho-Oncology* (n=2142), *Support Care Cancer* (n=1737), *Journal of Pain and Symptom Management* (n=1670), and *Cancer – American Cancer Society* (n=1451).

**Table 5:** Top 10 most highly represented journals publishing in this area.

Sources	Articles
Palliative & Supportive Care	109
Supportive Care in Cancer	73
Current Opinion in Supportive and Palliative Care	62
Indian Journal of Palliative Care	48
BMJ Supportive & Palliative Care	42
Journal of Pain and Symptom Management	35
Psycho-Oncology	35
BMC Palliative Care	28
Palliative Medicine	28
Cancer	25

**Table 6:** Top 10 cited journals publishing in this area.

Sources	Citations
Journal of Clinical Oncology	3977
Psycho-Oncology	2142
Support Care Cancer	1737
Journal of Pain and Symptom Management	1670
Cancer-American Cancer Society	1451
Annals of Oncology	1079
New England Journal of Medicine	906
Palliative Medicine	876
Lancet Oncology	824
Journal of Palliative Medicine	808

### 3.5 Author analysis

**Figure 5:**
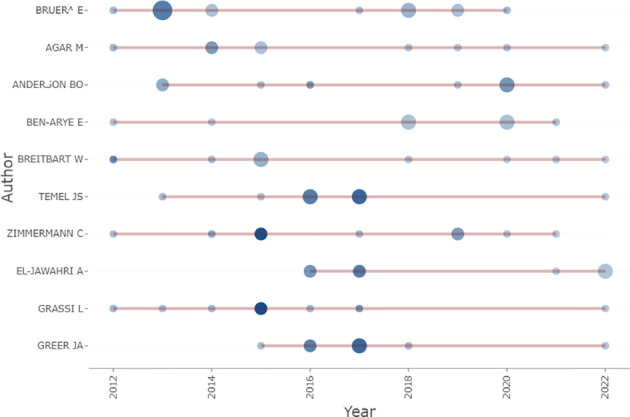
Top 10 most relevant authors’ production in the field of palliative care in breast cancer. (red line: publications start and end time, bubble size: the publications’ number, color intensity: number of citations of publications in that year).

**Figure 6:**
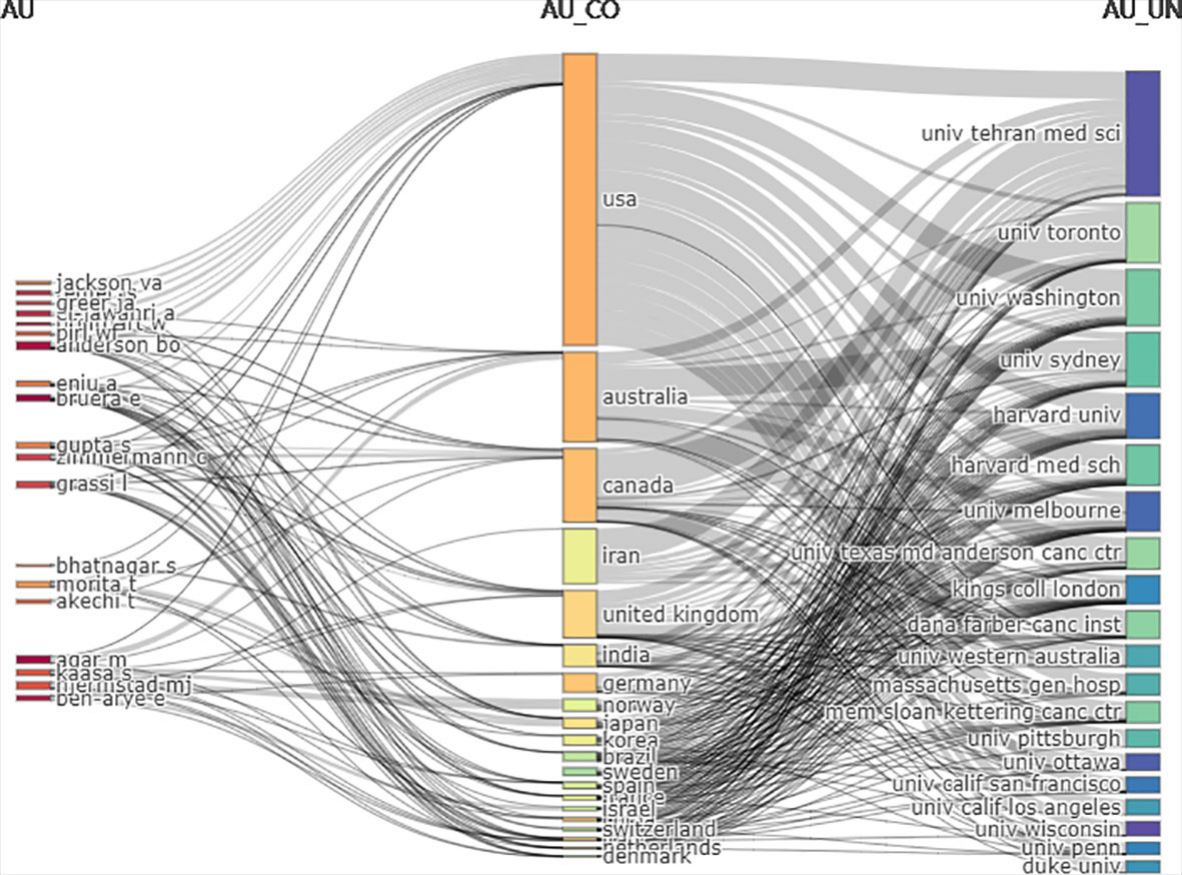
Three-fields plot of the countries (the middle field), the top authors (the left field), and the top affiliations (the right field) and their relationships in breast cancer palliative care.

A total of 8524 authors contributed to the 1529 publications including 61 who were the sole author of an article and 8463 as part of multi-author articles. The average number of authors per document was 5.57, and the average number of co-authors per document was 6.75. E. Bruera was the most productive author, contributing 16 publications (representing 1.05% of the total), followed by M. Agar, B.O. Aederson, E. Ben-Arye, W. Breitbart, A. El-Jawahri, J.S. Temel, and C. Zimmermann, who each contributed 9 publications (0.59% of the total). Authors who were more prolific had greater importance in the field, as shown in **Figure 5** where the size of the dot reflects the number of publications and the color shade corresponds to the total number of citations per year. E. Bruera had the most publications in the first 2 years, whereas M. Agar and C. Zimmermann maintained a stable output. The H index, which is a metric that combines the total number of publications (ie, productivity) and number of citations (ie, quality of those publications), provides an estimate of a researcher’s overall scientific productivity (21). E. Bruera had the highest H index at 10, followed by J.S. Temel (H index=8), B.O. Anderson (H index=7), L. Grassi (H index=7), J.A. Greer (H index=7), C.H. Yip (H index=7), and C. Zimmermann (H index=7); 19 of the most productive authors had an H index >5. Given that researchers have different qualifications, the M index can compensate for the defect of H index and serves as a remedy for correcting the H index for time (22). E. Bruera had the highest M index at 0.909, followed by J.A. Greer (M index=0.875), J.S. Temel (M index=0.8), B.O. Anderson (M index=0.7), M. Agar, L. Grassi, C.H. Yip, and C. Zimmermann (M index=1.167) (**Table 7**). The publications were analyzed with three-field plots that illustrated the relationship between the top authors, the top countries and the top affiliations (**Figure 6**).

**Table 7:** Top 10 contributing authors arranged in descending order of H index in the field of palliative care in breast cancer.

Name	H-index	G-index	M-index
Bruera E	10	16	0.909
Temel JS	8	9	0.8
Agar M	7	8	0.636
Anderson BO	7	9	0.7
Grassi L	7	8	0.636
Greer JA	7	8	0.875
Yip CH	7	7	0.636
Zimmermann C	7	9	0.636
Akechi T	6	7	0.6
Breitbart W	6	7	0.545

### 3.6 Keyword analysis

**Figure 7:**
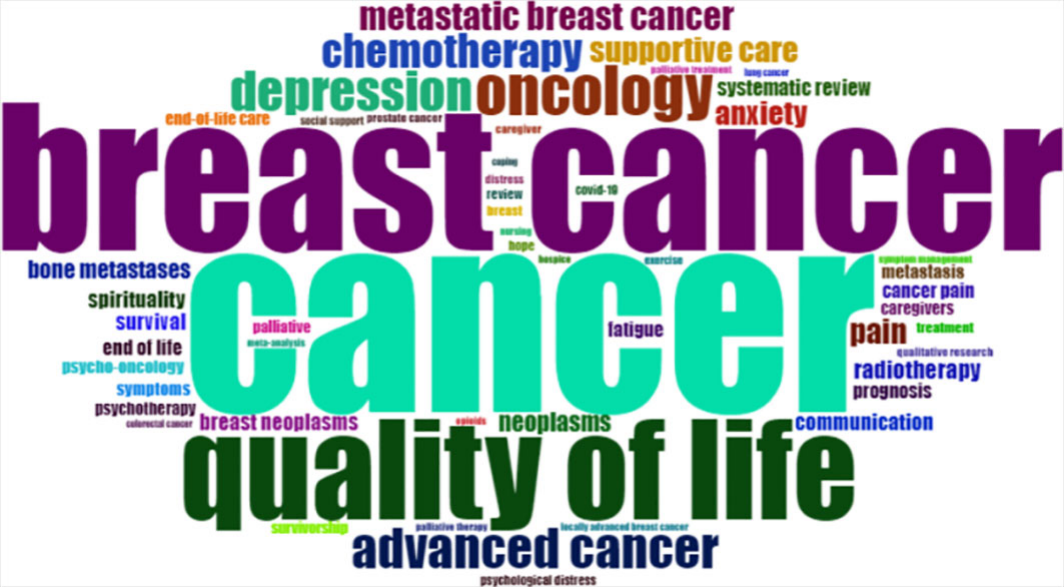
Word cloud of the most frequent author’s keywords in palliative care in breast cancer. (font size: frequency of occurrence).

**Figure 8:**
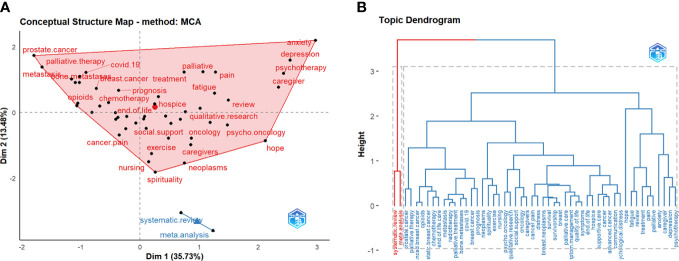
The most frequent author’s keywords in palliative care for breast cancer. **(A)** The Conceptual structure map. **(B)** The topic dendrogram (height: the distance between clusters or keywords).

We performed a keyword analysis of the 1529 publications with Bibliometrix and VOSviewer. **Table 8** lists the most frequent author keywords; only 19 of the 3424 author keywords extracted from the dataset reached the preset threshold (ie, appeared at least 25 times). Keywords related to palliative care for breast cancer included “quality of life”, “oncology”, “supportive care”, “communication”, “depression”, “advanced cancer”, and “chemotherapy”, highlighting the research hotspots. A word cloud was used to visualize frequently used keywords (**Figure 7**), with the font size reflecting the frequency of their occurrence. The most common author keywords were separated into 2 categories using multidimensional scaling methods (**Figure 8A**). The two dimensions of the graph represent the average location of publications that included each keyword, and the midpoint of the graph represents the center of the field of palliative care for breast cancer research. The red cluster contains most high-frequency keywords whereas the blue cluster contains the 2 remaining keywords. **Figure 8B** shows a dendrogram of research on palliative care for breast cancer as another form of the keyword conceptual map that also classified the keywords into 2 categories. The height of the connecting line in **Figure 8B** had the same average value as the distance between the keywords or clusters in **Figure 8A**. Each color in the tree describes a partition, and keywords connected by high connection lines represent the psychological aspects of breast cancer patients, which is a hotspot in palliative care for breast cancer research.

**Table 8:** Most frequent keywords in the field of palliative care in breast cancer.

Keyword	Occurrences
palliative care	304
cancer	290
breast cancer	226
quality of life	153
oncology	76
depression	63
advanced cancer	63
chemotherapy	53
metastatic breast cancer	44
supportive care	39
pain	38
anxiety	37
neoplasms	30
radiotherapy	29
bone metastases	28
communication	26
spirituality	26
survival	26
systematic review	25

**Figure 9:**
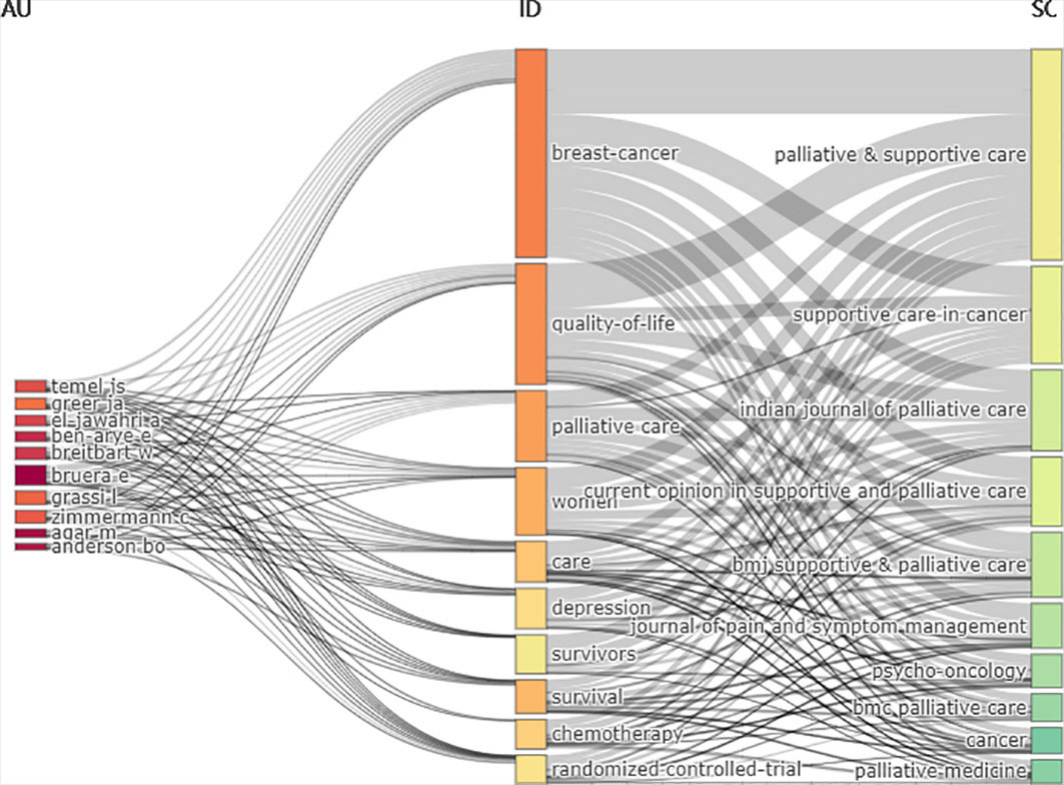
Three-fields plot of the most frequent keywords plus (the middle field), the top authors (the left field), and the top publication sources (the right field) and their relationships in palliative care for breast cancer.

Keywords plus are words or phrases that frequently appear in the titles of references cited by a retrieved article but not in the title of the article itself (23). The publications were analyzed with three-field plots that illustrated the relationship between the most frequent keywords plus, most prolific authors, and top publication sources (**Figure 9**). These 3 datasets constitute the middle, left, and right fields, respectively, of the plots. The height of each rectangle in the figure represents the sum of links from keywords plus, authors, or sources based on their co-occurrence. E. Bruera and W. Breitbart used most of the popular keywords plus in their publications; the most common keywords plus were also found in the 10 most highly represented journals, including “quality of life”, “depression”, and “care”, indicating that attention to breast cancer patients’ quality of life as well as anxiety and depression were the focus of many studies in the field.

## 4 Discussion

There has been growing research interest in improving breast cancer care by incorporating palliative care into standard clinical management programs. However, there have been few comprehensive, in-depth studies on palliative care interventions in breast cancer. In this study we performed a bibliometric analysis of publications in this research area from 2012 to 2022 in order to identify current and future research trends. In the examined time frame, there were 1529 publications on palliative care for breast cancer. The top 5 most-cited publications synthesized and summarized the existing data, highlighting the importance of palliative care for improving patients’ quality of life and revealing possible directions for future research. De Moor et al. (24) showed that palliative care interventions were associated with increased quality of life for patients. Breast cancer management strategies focused on early detection should also target access to palliative care; however, we found that there was no consensus on the optimal timing of palliative care interventions, and it remains unclear whether concurrent palliative and oncologic treatments and symptom control strategies for patients living for long periods of time with advanced disease (eg, stage IV breast cancer) can improve patients’ physical and psychological wellbeing in their last years of life.

The incidence of breast cancer is expected to increase in the future, which will cause further strain to healthcare systems worldwide (18). Most countries with a high GDP have implemented effective interventions, but the studies by Fitzmaurice et al. (18), Ginsburg et al. (20), and Knaul et al. (25) indicate that the need for palliative care is especially great for breast cancer patients in low- and middle-GDP countries, who have little or no access to such services. In our analysis, we found that low- and middle-GDP countries had a lower publication output and total connectivity strength. These countries require sustainable investments in all aspects of cancer management from prevention to care, as well as the development of high-quality population-based cancer registries. Although many studies examined differences in breast cancer burden, survival, and access to palliative care between high- and low-GDP countries, there were few that focused on how insufficiency of medical resources affects the delivery of palliative care, highlighting a gap in knowledge that warrants further investigation.

Keywords related to palliative care included “quality of life”, “oncology”, “supportive care”, “communication”, “pain”, “anxiety”, “depression”, “advanced cancer”, “chemotherapy”, and “spirituality”, which indicated the main focus of research on palliative care for breast cancer. The results of the keywords analysis were consistent with the subject domain findings. The keywords “depression”, “pain”, “anxiety” and “spirituality” reflected the role of psychological care as a form of palliation in breast cancer patients; 279 publications focused on this topic, but there was little information on the relationship between palliative care and depression in breast cancer patients. Therefore, it remains to be determined the significant degree of palliative care in reducing the anxiety and depression of breast cancer patients.

There are some limitations to our study. First, because of software limitations, it was difficult to merge the results from multiple databases and we searched only Web of Science, which does not cover all journals in every discipline. We also restricted the language of publication to English, which excluded some articles. On the other hand, Web of Science is the most widely used database for scientometric analyses and has some useful and easy-to-use functions such as “Analyze results” and “Create citation report”. Most bibliometrics software programs can identify Web of Science formats. Although it is not possible to obtain complete information about the article such as the country of the author, this can be circumvented by performing a manual search of authors. As the criteria for article selection was subjective, the opinions of multiple experts were required to screen publications. Second, the rapid progress in palliative care in recent years limited the timeliness of the analysis. Third, the software used to analyze the results had certain shortcomings. For example, VOSviewer’s co-citation analysis of authors from Web of Science included only the first author of the article. Additionally, some of the most cited articles were multidisciplinary and covered a broad range of topics, resulting in low specificity in the specific research area of palliative care for breast cancer. Furthermore, some of the articles were published many years ago and their conclusions may not reflect the current state of the knowledge in the field, thereby undermining their citation value. We emphasize that publications represent the current focus of the academic community but do not necessarily reflect the application or impact of palliative care in actual practice. Nonetheless, the results of our analyses suggest future directions for research on palliative care in breast and other cancers.

## 5 Conclusion

In the past decades, palliative care has transitioned from a concept to clinical application with the growing awareness of the importance of humanistic care focused on the psychological state of patients. This study analyzed articles on the value of palliative care for breast cancer published from 2012 to 2022. The results revealed collaboration networks of contributing countries, institutions, journals, and authors as well as landmark studies, research hotspots, and future research directions. Health services and supportive care as well as pain and symptom management were the main research topics. The US has been a major contributor to the field of breast cancer palliative care; and University of Toronto and E. Bruera have made significant contributions. *Palliative & Supportive Care* was the journal with the largest number of relevant publications. Important research directions for the future include the degree to which palliative care can reduce anxiety and depression in breast cancer patients; the optimal timing of palliative care interventions; and resolving the disparity between available medical resources and the effective delivery of palliative care. In view of the enormous burden of cancer on healthcare resources worldwide, greater recognition of the benefits of palliative care can improve cancer management and potentially lead to better outcomes for patients.

## Data availability statement

The original contributions presented in the study are included in the article/supplementary material. Further inquiries can be directed to the corresponding authors.

## Author contributions

YS and HT contributed to study conception and design. All authors contributed to the development of the study protocol. XQ and YF provided guidance on the use of analysis applications and statistical analyses. JW extracted the data from the Web of Science, performed the statistical analysis, and was a major contributor to manuscript writing. YX was involved in the interpretation of the study findings. JZ and YZ critically revised the article. All authors read and gave final approval for the submitted version. All authors contributed to the article and approved the submitted version.
